# Folate-modified erythrocyte membrane nanoparticles loaded with Fe_3_O_4_ and artemisinin enhance ferroptosis of tumors by low-intensity focused ultrasound

**DOI:** 10.3389/fonc.2022.864444

**Published:** 2022-08-10

**Authors:** Xingyue Wang, Peng Li, Xiangxiang Jing, Yun Zhou, Yongfu Shao, Min Zheng, Junrui Wang, Haitao Ran, Hailin Tang

**Affiliations:** ^1^ Department of Ultrasonography, Xiangyang Central Hospital, Affiliated Hospital of Hubei University of Arts and Science, Xiangyang, China; ^2^ Chongqing Key Laboratory of Ultrasound Molecular Imaging, Institute of Ultrasound Imaging, Second Affiliated Hospital, Chongqing Medical University, Chongqing, China; ^3^ Department of Diagnostic Ultrasoundand Echocardiography, Tongde Hospital of Zhejiang Province, Hangzhou, China; ^4^ Department of Ultrasound, Hainan General Hospital (Hainan Affiliated Hospital of Hainan Medical University), Haikou, China; ^5^ Department of Ultrasound, the International Peace Maternity and Child Health Hospital, School of Medicine, Shanghai Jiao Tong University, Shanghai, China

**Keywords:** cell membrane biomimetic, artemisinin, low-intensity focused ultrasound, controlled drug release, ferroptosis

## Abstract

To overcome the challenges of the low efficiency of artemisinin (ART) in anticancer therapy due to its poor water solubility and poor bioavailability, we constructed folate (FA)-modified erythrocyte membrane (EM)-camouflaged poly (lactic-co-glycolic acid) (PLGA) nanoparticles (NPs) (PFH/ART@PLGA/Fe_3_O_4_-eFA). Specifically, the inner core of these NPs is mainly composed of phase-changeable perfluorohexane (PFH), magnetic Fe_3_O_4_ and ART. *In vitro* experiments showed that the prepared PFH/ART@PLGA/Fe_3_O_4_-eFA was readily taken up by 4T1 cancer cells. PFH/ART@PLGA/Fe_3_O_4_-eFA was exposed to low-intensity focused ultrasound (LIFU) irradiation to induce PFH phase transition and NPs collapse, which promoted the release of ART and Fe_3_O_4_. After LIFU irradiation, the proportion of dead 4T1 cells, the level of reactive oxygen species (ROS) and the concentration of intracellular Fe^2+^ ions in the PFH/ART@PLGA/Fe_3_O_4_-eFA group were much higher than those in the other group, indicating that the synergistic effect between the intracellular Fe^2+^ ions and the released ART played a critical role in tumor cell ferroptosis by enhancing ROS generation *in vitro*. We demonstrated that FA-modified EM NPs could enhance the targeting and accumulation of the NPs at the tumor site *in vivo*. After LIFU irradiation at 3 W/m^2^ for 7 min, tumor growth was completely suppressed through FA-modified EM NPs collapse and the release of ART and Fe_3_O_4_, which exerted synergistic effects in inducing tumor ferroptosis. Because of these characteristics, these NPs are considered as a promising approach for the delivery of drugs with poor water solubility for efficient cancer therapy.

## Introduction

ART and its derivatives are among the most important and effective antimalarial drugs in clinical use. Moreover, they feature distinct antitumor activity against various types of cancer cell lines. However, their use in the clinic is also limited by their poor water solubility and poor bioavailability, which is one of the main problems that causes their low anticancer activity (1). To overcome the aforementioned limitations associated with the use of ART and its derivatives for the treatment of cancer, they have been incorporated into nanoparticles (NPs) (2), which are highly effective in the field of drug delivery (3). However, studies have found that NPs, which are usually larger than 100 nm in diameter, are often captured by the body’s immune system or reticulo endothelial system (RES) *in vivo* (4). After intravenous injection, NPs still suffer from passive immunological clearance, which are mainly concentrated in organs rich in reticulo endothelial cells, especially in the liver and spleen, and in the bone marrow, causing the unsatisfactory targeting abilities. These characteristics lead to decreased working concentrations of NPs intarget organs (5–7).

In recent years, biomimetic approaches using natural cell membranes, including EM, to camouflage NPs have attracted particular attention because of their intrinsic biocompatibility, biodegradability, and nonimmunogenicity (8). EM has been used as a desirable carrier to deliver various bioactive compounds. Notably, EM-coated nanoformulations have been widely used in antitumor research and have enhanced antitumor effects (9, 10). Moreover, studies have demonstrated that EMs grafted with folic acid (FA) successfully performed active tumor-targeting properties (11; 12). The advantages of the use of EMs grafted with FA as carriers for loading NPs include a)the ability to escape the immune system and maintain long-term circulation; b) the intrinsic desirable biocompatibility and biodegradability; and c) the ability to efficiently target tumors (13). However, FA-modified EM coatings cannot be efficiently degraded in the tumor microenvironment, which greatly influences the release of drugs from NPs (14).Therefore, there is an urgent need to develop a strategy for efficiently constructing a stimulus-responsive EM–derived drug delivery nanosystem. External stimuli, such as photodynamic therapy (15), photothermal stimulation (16), ultraviolet irradiation (17), laserirradiation (18), and sonodynamic therapy (19), have been effectively used to activate the release of antitumor drugs from EM-derived NPs into the tumor site.

In our previous studies (20), the ultrasound irradiation duration and power of LIFU were tested and selected to control the release of drugs from NPs. The thermal effect generated by LIFU induced the liquid−gas phase transition of PFH in the NPs to generate PFH microbubbles. Various ultrasound energies were introduced to induce the phase transition of PFH and the microbubble collapse of NPs *in vitro*. Our research team provided a new strategy for efficient ultrasound-triggered delivery of chemotherapy by NPs with programmable LIFU that is capable of achieving on-demand drug release (20). Based on previous studies, we present the construction of a phase-changeable drug delivery system involving PLGA cloaked with FA-modified EMs; this system allows the programmable LIFU-triggered release of drugs and significantly enhances anticancer drug delivery. The as-designed EM-NPs consist of five individual parts. First, EMsallow the escape of the immune system and maintain long-term circulation due to their inherent biocompatibility and biodegradability. Second, FA is covalently bound onto the outer shell of the EM-NPs to target transportation to and accumulation in the tumor tissue. Third, liquid-gas phase-changeable PFH is encapsulated into the core of the EM-NPs, and it can be vaporized by LIFU activation. Fourth, ART, which is a natural drug with potent anticancer activities but poor water solubility, is loaded into the EM-NPs. Fifth, super paramagnetic Fe_3_O_4_ NPs is integrated into the shell of the EM-NPs to provide the NPs with the ability to be detected by T2-weighted magnetic resonance imaging (MRI) and to enhance the antitumor efficacy of ART.

**Scheme 1:**
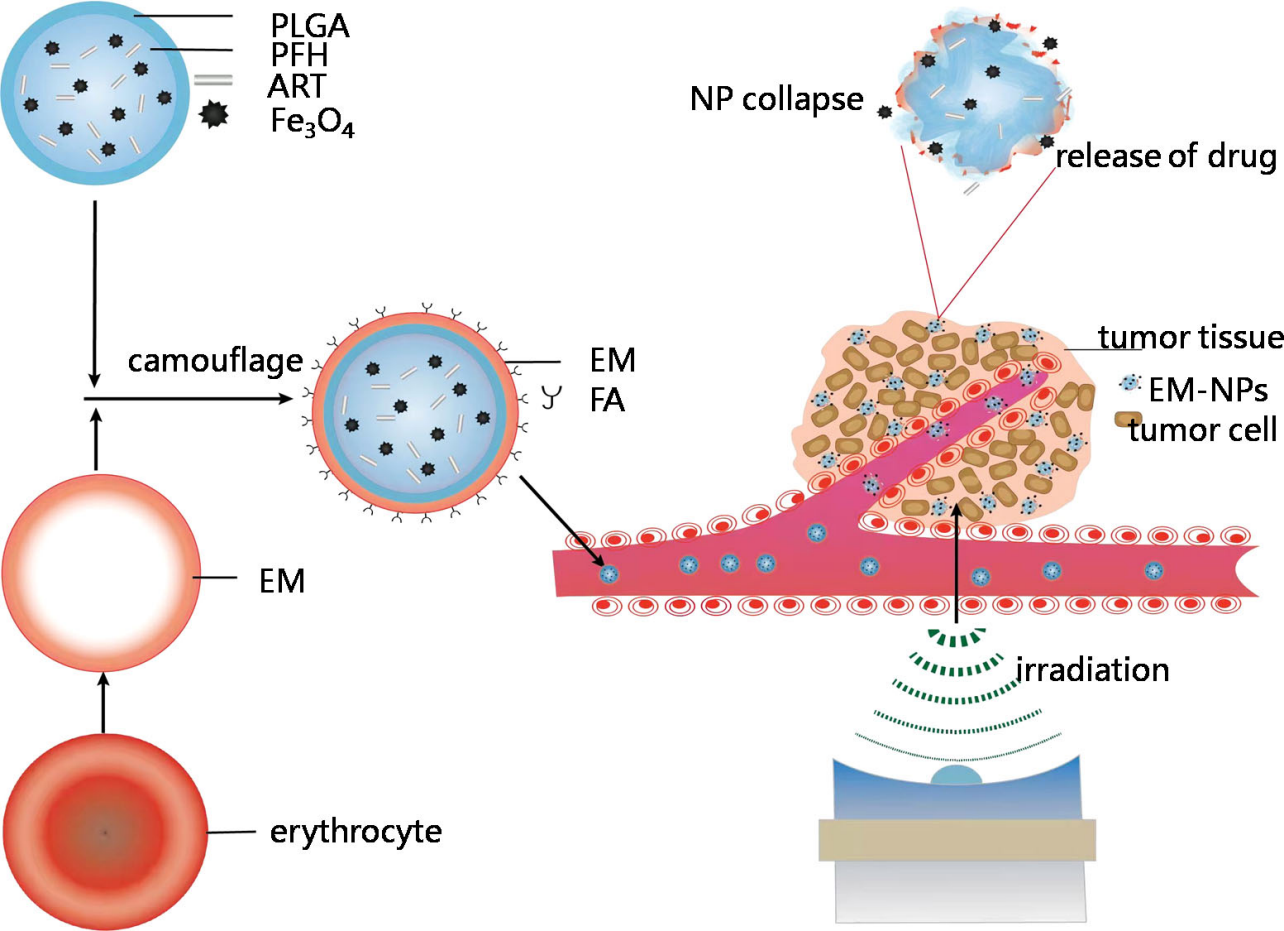
Schematic showing construction and delivery of multifunctional EM-NPs and release of ART and Fe_3_O_4_ through NP collapse in tumor site by LIFUirradiation.

This nanotheranostic agent is based on PLGA-NPs with temperature-responsive PFH at their core, super paramagnetic Fe_3_O_4_ NPs in their shell, the anticancer drug ART encapsulated inside, and FA decorated on the EMs; these NPs were mechanically transformed into vesicles and reconstructed on a FA-modified EM encapsulating PLGA(designated as PFH/ART@PLGA/Fe_3_O_4_-eFANPs).This novel multifunctional EM-NP can serve as a promising candidate for further studies on cancer therapy because of its favorable features that allow immune escape, efficient targeting, tumor accumulation, temperature-responsive phase transition, stimulus-responsive drug delivery and LIFU irradiation-mediated regulation of drug release for efficient tumor therapy. It is possible that the low pH value in malignant tumor cells destabilizes the binding of iron to transferrin and allows iron to escape from transferrin Fe^3+^ to Fe^2+^ after more Fe_3_O_4_ enters the tumor cells (21).The designed strategy could result in more ferroptosis in the cancer cells by synergistic effects of the cytotoxicity of ART and increased intracellular levels of Fe^2+^ ions (6; 22). The design idea of this study is shown in the **Scheme 1**.

## Materials and methods

### Materials

Carboxyl-modified PEGylatedpoly (lactic-co-glycolic acid) (lactide: glycolide =650:50, PLGA = 40,000 Da MW, PEG = 10,000 Da MW) (PLGA-PEG10,000-COOH) was obtained from Jinan Daigang Biomaterial Co., Ltd. (Shandong, China). Oleic acid-coated super paramagnetic iron oxide (SPIO) nanoparticles (NPs) (d = 20 nm, 28 mg/mL) were purchased from Ocean Nano Tech, Inc. (Arkansas, USA).Perfluorohexane (PFH, boiling point of 56°C), poly(vinyl alcohol)(PVA, 25,000 MW), the fluorescence dye 1,1’-dioctadecyl-3,3,3’,3’tetramethylindocarbocyanine perchlorate (DiI),2,7-dichlorodihydrofluorescein-diacetate (DCFH-DA),artemisininand 2-(4-amidinophenyl)-6-indolecarbamidine dihydrochloride (DAPI) were obtained from Sigma–Aldrich Co.(Missouri, USA). All the reagents used in this work were of analytical grade.

### Preparation of EMs and EMs with ligands embedded

EM-derived vesicles were prepared using hypotonic hemolysis as previously reported (23). The final EM product was stored in deionized water at 4°C for future use. The double emulsion method was used to generate PLGA-based nanoemulsions according to our previously described experimental method (24).

### Preparation of EM-Coated PLGA-NPs

The EM-coated PLGA-NPs were prepared by the liposome extrusion method with an Avanti mini-extruder (Avanti PolarLipids). To form erythrocyte ghosts with ligands embedded, the light pink solution was incubated with distearoyl phosphoethanolamine-polyethylene glycol-FA for 30 min. briefly, 1 mg PLGA-NPs were mixed with 23 μL of EMs from whole blood. The resultant mixture was subsequently extruded nine times through a 400-nm polycarbonate porous membrane using an Avanti mini-extruder to yield EM-coated PLGA-NPs or FA-EM-coated PLGA-NPs.

### Characterization of EM-NPs

The hydrodynamic diameter and zeta potential of the NPs suspended in PBS were measured by dynamic light scattering (DLS, Malvern Instruments Ltd., UK). The stability of the EM-NPs in PBS was monitored by DLS. The proteins retained on the EM-NPs compared with those retained on natural EMs were observed by sodium dodecyl sulfate-polyacrylamide gel electrophoresis(SDS–PAGE).CD47 expression on the EM-NPs was measured by Western blotting.

The encapsulation efficiency and loading capacity of ART were determined by measuring the free ART in the supernatant from the EM-coated PLGA NPs by centrifugation at 12,000 rpm for 30 min. The *in vitro* release profiles of the NPs were determined by suspending FA-EM-PLGA in release medium (PBS with 20% ethyl alcohol). Samples (2 mL) were withdrawn at 30 min interval initially and later every 2 h for 48 h by replacing with equal amount of PBS to maintain the sink condition. The levels of free ART in the supernatant were measured by a UV1700 spectrophotometer (Shimadzu, Japan) at 292 nm. The ART encapsulation efficiency (EE) and ART loading capacity (LC) were calculated as follows:

Artemisinin Encapsulation Efficiency (EE) = (Total artemisinin − Free artemisinin)/Total artemisinin× 100%.

Artemisinin Loading Capacity (LC) = (Total artemisinin − Free artemisinin) NPs weight× 100%

### Cell culture and nude mouse model

The breast cancer cell line 4T1 and macrophage cell line RAW264.7 were obtained from Chongqing Medical University. The two cell lines were cultured in Roswell Park Memorial Institute 1640 supplemented with 10% fetal bovine serum (FBS) and 1% penicillin/streptomycin at 37°C in 5% CO_2_. Female Balb/c mice (4-6 weeks, 15-19 g)were purchased from and housed at the Animal Center of Chongqing Medical University. To establish the tumor model, 4T1 cells were detached with trypsin (0.125% v/v) and resuspended in fresh sterile PBS solution. Then, the cells (1× 10^6^ cells per mouse) were subcutaneously injected into the left flanks of the mice. Tumor-bearing mice were used for additional *in vivo* experiments when the tumors reached a volume of 80 mm^3^.

### *In vitro* and *in vivo* biocompatibility studies

To measure cytotoxicity*in vitro*, the CCK-8 assay was used.4T1 cells and RAW264.7 cells were seeded in 96-well plates (1×10^4^cells per well) in medium supplemented with 10% FBS and 1% penicillin/streptomycinand incubated overnight to allow the cells to adhere. Fresh medium containing PFH/ART@PLGA/Fe_3_O_4_NPs and PFH/ART@PLGA/Fe_3_O_4_-eFA NPs at different concentrations (0, 2, 4, 6, 8, and 10 mg/mL) were added to replace the previous medium and coincubated with the cells for 24 h. Finally, the absorbance values were measured at 460 nm by a microplate reader (BIO-TEK EL × 800, USA), and the cell viability was calculated using the following equation: [(OD NPs – OD blank)/(ODwithout NPs – OD blank) × 100%].

To evaluate toxicity *in vivo*, healthy female Balb/cmice (5 weeks, ~16 g) were randomly divided into three groups (n = 3), which were then intravenously injected with PFH@PLGA/Fe_3_O_4_-eFA NPs. The dose of the NPs was 1.6 mg permouse. On days 0, 1, 7, and 28 after injection, blood and serum were collected from each mouse, and hematological analysis was conducted to evaluate acute toxicity. At Day 28 after injection, the mice were sacrificed, and the major organs (heart, liver, spleen, lungs and kidneys) were harvested for pathological sectioning and hematoxylin and eosin (H&E) staining to evaluate the long-term biosafety of the NPs.

### *In vitro* and *in vivo* targeting studies

4T1 cells were seeded in confocal laser scanning microscope (CLSM) culture dishes (1×10^5^ cells) with medium supplemented with 10% FBS and 1% penicillin/streptomycin and incubated overnight to allow the cells to adhere. PFH@PLGA/Fe_3_O_4_-eFA NPs, PFH@PLGA/Fe_3_O_4_-e NPs and PFH@PLGA/Fe_3_O_4_NPs were dispersed in 1 mL of fresh serum-free medium, and each solution was added to separate dishes. After coincubation for 2 h, the cells were washed three times with PBS and fixed with 4% paraformaldehyde for 10 min. Then, DAPI(10 μg/mL) was used to stain the nuclei of the cells for 10 min, followed by washing with PBS. Finally, the cells were observed by CLSM. For comparison, RAW264.7 cells were seeded as described above. For distribution of NPs *in vivo*, tumor-bearing nude mice were divided into a targeted group and a nontargeted group, and these groups received intravenous administration of DiR-labeled PFH/ART@PLGA/Fe_3_O_4_-eFANPs and DiR-labeled PFH/ART@PLGA/Fe_3_O_4_ NPs(1.6 mg per mouse), respectively. The bio distribution of the nanoparticles was evaluated by alive florescence imaging system at different time points (preinjection and 6 h, 12 h, 24 h and 48 h postinjection).

### *In vitro* liquid–gas phase transition by LIFU

The NPs were added to an agarose gel phantom (3% agarose). Then, the LIFU transducer (1 MHz; acoustic tensity: 3 W/cm^2^; duty cycle: 50%; Chongqing Medical University, P. R. China) was placed perpendicular to the surface of the wells in the gel. The NPs were exposed to LIFU irradiation for duration of 1-10 min. Harmonic ultrasound-imaging signals of the microbubbles after formation from nanodroplets were observed in real-time with a MyLab 90 ultrasound diagnostic system (Esaote, Italy). The average ultrasound intensity was determined by DFY software (Chongqing Medical University, P. R. China).The liquid–gas phase transition of PFH in the NPs was observed with an optical microscope (Olympus IX53, Canada).

### *In vitro* therapy

To evaluate the concentration-dependent cytotoxicity of the NPs*in vitro*, 4T1 cells were seeded in a 96-well plate (8×10^3^cells per well). Then, the cells were coincubated with different treatments (PBS,LIFU (U), PFH@PLGA/Fe_3_O_4_, PFH/ART@PLGA, PFH/ART@PLGA/Fe_3_O_4_, PFH/ART@PLGA/Fe_3_O_4_+U,or PFH@PLGA/Fe_3_O_4_-eFA+U). For the ultrasound-treated groups, cells in fresh medium were exposed to an ultrasonic wave at 3 W/cm^2^ for 5 min and incubated for an additional 24 h. Finally, the relative cell viabilities in all the groups were evaluated by using the standard CCK-8 assay. *In vitro* cell death analysis was conducted on the following seven groups as above. 4T1 cells seeded in 6-well plates at a density of 1×10^5^ cells per well and were coincubated with different NPs at 8 mg/mL. Finally, cell death was measured by flow cytometry.

### Investigation of intracellular Fe^2+^ concentration and ROS production

To detect Fe^2+^ ions generation in 4T1 breast cancer cells, CLSM measurement was performed. 4T1 cells were seeded in confocal dishes(1 × 10^5^ cells/mL), cultured for 24 h, divided into 7 groups (PBS, LIFU, PFH@PLGA/Fe_3_O_4_, PFH/ART@PLGA, PFH/ART@PLGA/Fe_3_O_4_, PFH/ART@PLGA/Fe_3_O_4_+U, PFH@PLGA/Fe_3_O_4_-eFA+U). For the ultrasound-treated groups, cells in fresh medium were exposed to an ultrasonic wave at 3 W/cm^2^ for 5 min. After cells were incubated for 6 h, the culture medium was removed and cells were washed three times with PBS. Then FerroOrange (1 µM, an intracellular Fe^2+^ ions probe, Ex: 543 nm, Em: 580 nm) dispersed in serum-free medium was added to the cells, and incubated for another 30 min in a 37°C 5% CO_2_ incubator. Finally, the fluorescence images of the cells were detected by inverted fluorescence microscope.

For ROS production inside living cells, a fluorescence microplate using 2′,7′-dichlorofluoresceindiacetate (DCFH-DA) was used as the fluorescence probe, 4T1 cells were seeded in 6-well plates at a density of 1 × 10^5^ per well. 24h after seeding, the culture wells were randomly divided into 7 groups (PBS, LIFU, PFH@PLGA/Fe_3_O_4_, PFH/ART@PLGA, PFH/ART@PLGA/Fe_3_O_4_, PFH/ART@PLGA/Fe_3_O_4_+U, PFH@PLGA/Fe_3_O_4_-eFA+U). After cells were administered with above treatments and stained with DCFH-DA (10 μM) at 37°C for another 30min, the culture medium was discarded and the cells were washed with PBS three times. Then the fluorescence was recorded with inverted fluorescence microscope.

### *In vitro* and *vivo* imaging

For *in vivo* ultrasound imaging, the ultrasound transducer (1 MHz;acoustic density: 3 W/cm^2^; duty cycle:50%; Chongqing Medical University, P. R. China) was fixed above the tumor in contact with a gel interface coupled with an Anextra 1.5-cm-thick gel bag. The fundamental and harmonic imaging signals of the bubbles were observed in real-time with a MyLab90 ultrasound diagnostic system (Esaote, Italy). The average ultrasound intensity values were measured by DFY software (Chongqing Medical University, P. R.China).For MRI imaging, to determine the ability of PFH/ART@PLGA/Fe_3_O_4_-eFA and PFH/ART@PLGA/Fe_3_O_4_ NPs as detected by MRI *in vivo*, T2 images and the T2 relaxation rate of the tumor area were captured at different time points (0, 3, 6, 12 and 24 h) after intravenous injection of the NPs. The T2WI parameters were set as follows: the repetition time was 8000 ms, the echo time was 85 ms and the slice thickness was 1.50 mm using a 3.0 T MRI scanner (MAGNETOM Prisma, SIEMENS, Germany).

### *In vivo* antitumor effect

BALB/c nude mice received 4T1 cellxenografts and were randomly divided into groups. The control group was treated with saline. The ultrasound groups were sonicated by LIFU as described above. Samples (0.2 mL) were administered *via* the tail vein. The tumor volume was monitored by caliper every three days and calculated with the following equation: Volume=0.5×L×W^2^ (L and W are the length and width of the tumors, respectively). Histological and immunohistochemical experiments were carried out. Tumors were collected from the different groups and subjected to hematoxylin/eosin (H&E) staining. To assess tumor cell proliferation, immunohistochemical staining with a TdT-dependent UTP-biotin nick end labeling (TUNEL) assay kit was performed. Apoptosis was assessed using an apoptosis detection kit.

### Statistical Analysis

All results are shown as the mean values ± standard deviations. One-way ANOVA and Student’s t-test were adapted to analyze the data using SPSS version 21.0 software (IBM, Armonk, NY, United States). P< 0.05 was considered statistically significant.

## Results and discussion

### Biophysical characterization of PFH/ART@PLGA/Fe_3_O_4_-eFA

**Figure 1:**
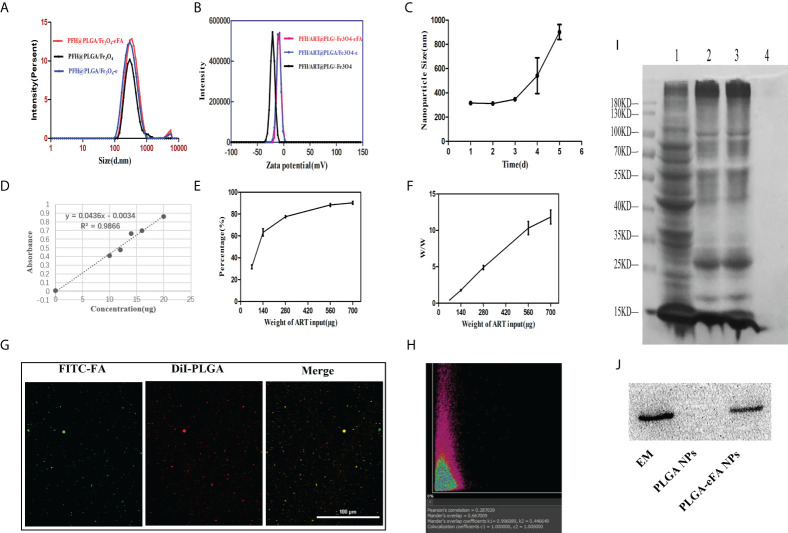
Biophysicalcharacterization of NPs. **(A)** Changes in the size of PFH/ART@PLGA/Fe_3_O_4_-eFA in PBS (pH 7.4) solution **(B)** Zeta potential of PFH/ART@PLGA/Fe_3_O_4_, PFH/ART@PLGA/Fe_3_O_4_-e, and PFH/ART@PLGA/Fe_3_O_4_-eFA **(C)** Size changes of PFH/ART@PLGA/Fe3O4-eFA in PBS (pH 7.4) solution containing with 10% FBS. **(D)** Corresponding relationship between the concentration of ART and absorbance optical density. **(E)** The drug encapsulation efficiency of different concentrations of ART. **(F)** The loading capacity of NPs with different concentrations of ART. **(G)** CLMS images of PFH/ART@PLGA/Fe_3_O_4_-eFA. **(H)** The results of the binding efficiency of FA-modified EMs and PFH/ART@PLGA/Fe_3_O_4_ was further validated through a quantitative assay by flow cytometry. **(I)** SDS–PAGE results of different NPs (1 representserythrocyte, 2 represents EMs, 3 represents PFH/ART@PLGA/Fe_3_O_4_-eFA, and 4 represents PFH/ART@PLGA/Fe_3_O_4_). **(J)** Western blotting analysis of the expression of the membrane-specific protein marker CD47 in EMs,PFH/ART@PLGA/Fe_3_O_4_-eFA, and PFH/ART@PLGA/Fe_3_O_4_ (from left to right). All the error bars represent the standard deviations. n = 3.

The average diameter and Zeta potential of PFH/ART@PLGA/Fe_3_O_4_, PFH/ART@PLGA/Fe_3_O_4_-e,and PFH/ART@PLGA/Fe_3_O_4_-eFA NPs were in range of 298.1 ± 22.2 nm,315.3 ± 13.2 nm, and 319.3 ± 14.0 nm(Fig. 1A) and -20.4± 2.5mV, -8.3 ± 2.5mV, and -5.3 ± 2.6mV (**Figure 1A**), respectively. The effect of EM coating on the dynamic size and surface zeta potential of the PFH/ART@PLGA/Fe_3_O_4_ NPs was measured. The results indicated that the size of the NPs increased from 298 nm to 315 nm and the surface zeta potential of the NPs increased from -20.4 mV to -8.3 mV after EM coating. This may be related to the lower surface zeta potential of PLGA compared to EMs. These data verify the successful coating of PFH/ART@PLGA/Fe_3_O_4_ by EMs. Next, the effect of FA modification on the dynamic size and surface zeta potential of PFH/ART@PLGA/Fe_3_O_4_-e was measured. The results indicated that the size of the NPs increased from 315 nm to 319 nm and the surface zeta potential of the NPs increased from −8.3 mV of PB to −5.3 mV after FA modification (**Figure 1B**). FA molecules were modified on the surface of PFH/ART@PLGA/Fe_3_O_4_-e to endow them with the tumor-targeting ability. The average size of PFH/ART@PLGA/Fe_3_O_4_-eFA showed no obvious difference during incubation for 3days in PBS (**Figure 1C**), verifying that EM camouflage is beneficial to the high stability of PFH/ART@PLGA/Fe_3_O_4_-eFA in the bioenvironment. However, the average diameter of PFH/ART@PLGA/Fe_3_O_4_-eFA began to increase on the fourth day of incubation in PBS. We studied the encapsulation efficiency and loading capacity of ART. There was a good linear relationship between the absorbance optical density and the concentration of ART (**Figure 1D**). As shown in **Figure 1E**, when the ART concentration was 70,140, 280, 560 and 700 μg/mL, the ART encapsulation efficiency was 31.71%, 63.24%, 77.59%, 88.33% and 90.31%, respectively. As shown in **Figure 1F**, when the ART concentration was 70,140, 280, 560 and 700 µg/mL, the ART loading capacity of PFH/ART@PLGA/Fe_3_O_4_-eFA was 0.44%,1.79%,4.90%,10.31% and 11.83%,respectively.

The binding efficiency of the FA-modified EMs and PFH/ART@PLGA/Fe_3_O_4_ is crucial for escaping the immune system and targeting tumors. In the CLMS images, the red fluorescence is DiI packaged by PLGA, which indicates PFH/ART@PLGA/Fe_3_O_4_ (**Figure 1G**), and the green fluorescence is FITC-labeled FA (**Figure 1G**). As shown in **Figure 1G**, the yellow fluorescence was observed by CLSM, indicating the overlap of the red fluorescence that stains DiIand indicates PFH/ART@PLGA/Fe_3_O_4_ and the FITC green fluorescence staining of FA-modified EMs. These results show that FA-modified EMs were successfully connected to the surface of the PFH/ART@PLGA/Fe_3_O_4_ NPs. The Manders overlap coefficient was determined to assess the degree of colocalization or the relative fraction of overlap between the two fluorescence signals. In this study, the Mandersoverlap coefficient was 70.2%, according to quantitative analysis (**Figure 1H**). Furthermore, sodium dodecyl sulfate–polyacrylamide gel electrophoresis(SDS–PAGE) protein analysis indicated that PFH/ART@PLGA/Fe_3_O_4_-eFA had a protein profile similar to that of EMs alone, but PFH/ART@PLGA/Fe_3_O_4_ did not have a protein profile similar to that of EMs alone (**Figure 1I**); these results suggested the successful translocation of the EMs to the PLGA surface. CD47, an immunomodulatory protein expressed by erythrocytes and responsible for inhibiting macrophage uptake, was revealed to be expressed on the EMs. Further Western blotting analysis revealed that the EM ghost and PFH/ART@PLGA/Fe_3_O_4_-eFA had similar CD47expressionpatterns (**Figure 1J**).

### *In vitro* and *in vivo* biosafety of PFH/ART@PLGA/Fe_3_O_4_-eFA

**Figure 2:**
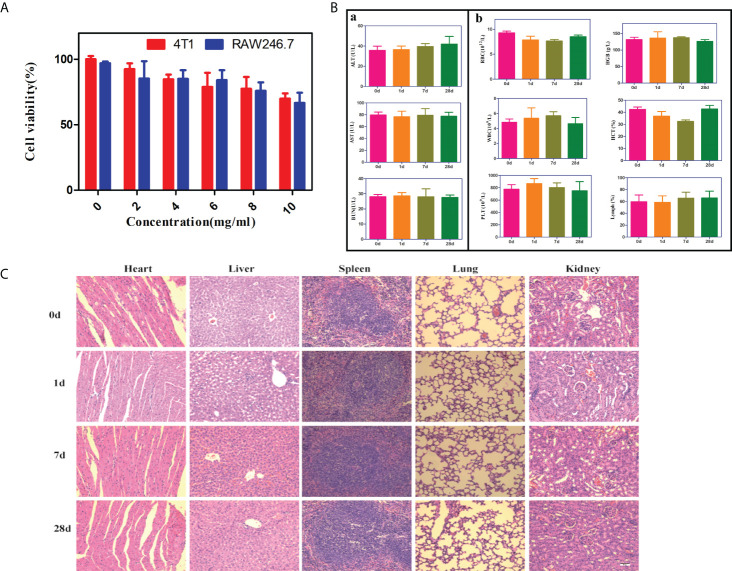
*In vitro* and *in vivo* biosafety of PFH/ART@PLGA/Fe_3_O_4_-eFA. **(A)** The biocompatibility of different concentrations of PFH/ART@PLGA/Fe_3_O_4_-eFA NPs was determined by a standard CCK-8 assay. **(B)** Routine blood and biochemical tests were performed at different times (0, 1, 7, 28d) after the injection of NPs. ①The changes of liver function (ALT and AST) and renal function (BUN) in mice.②The results of routine blood tests (WBC, RBC, HGB, HCT, PLT and MPV). **(C)** The H&E staining results of the liver, spleen, heart, lungs and kidneys of the mice at different time points (0, 1, 7, 28d) after the injection of PFH/ART@PLGA/Fe_3_O_4_-eFA (The scale bar is 50µm). All the error bars represent the standard deviations. n = 3.

We further investigated the cytotoxicity of PFH/ART@PLGA/Fe_3_O_4_-eFA *in vitro* by using the CCK-8 assay.4T1breast cancer cells and RAW246.7 cells were incubated with various NPs at different concentrations (0, 2, 4, 6, 8 and 10 mg/mL) for 24 h and then washed with PBS, and the cell viability was investigated 24 h after incubation. As shown in **Figure 2A**, the cell survival rate was greater than 80% when the concentration of PFH/ART@PLGA/Fe_3_O_4_-eFA was maintained lower than 8 mg/mL, indicating that there was no notable toxicity to 4T1 and RAW246.7 cells. Liver injury was evaluated by measuring the concentrations of ALT and AST. Kidney injury was evaluated by measuring the concentration of BUN. Routine blood tests were performed to measure the concentrations of WBCs, RBCs, HGB, HCT, PLT and MPV. The ALT, AST, BUN, WBC, RBC, HGB, HCT, PLT and MPV concentrations of the mice injected with 8 mg/mL PFH/ART@PLGA/Fe_3_O_4_-eFA NPs were not significantly different from those of the control mice (Day 0), proving that the PFH/ART@PLGA/Fe_3_O_4_-eFA NPs were nontoxic to the mouse liver, kidneys or blood at different time points (1, 7, 28d) (**Figure 2B**). To investigate the *in vivo* histocompatibility of PFH/ART@PLGA/Fe_3_O_4_-eFA NPs, representative H&E staining images were captured. The major organs, including the lungs, spleen, liver, heart, and kidneys, were collected at different time points (Day 0, 1,7,and 28) after the mice were intravenously injected with PFH/ART@PLGA/Fe_3_O_4_-eFA NPs, and the tissues were examined to compare histological changes. This examination proved that the PFH/ART@PLGA/Fe_3_O_4_-eFA NPs were not potentially toxic to biological organs (**Figure 2C**).

### *In vitro* 4T1 cell-targeting behavior of PFH/ART@PLGA/Fe_3_O_4_-eFA

**Figure 3:**
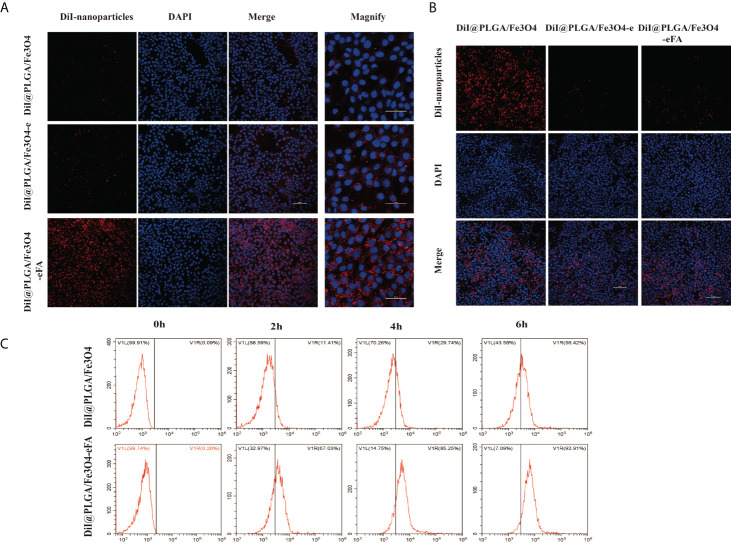
*In vitro* 4T1 cell-targeting behavior of PFH/ART@PLGA/Fe_3_O_4_-eFA. **(A)** CLSM images of 4T1 cells after incubation with PFH/ART@PLGA/Fe_3_O_4_, PFH/ART@PLGA/Fe_3_O_4_-e and PFH/ART@PLGA/Fe_3_O_4_-eFA NPs. From the left to the right, the images showDiI-labeled NPs, DAPI-labeled nuclei, the corresponding merged images and magnified images. **(B)**CLSM images of RAW246.7 cells after incubation with PFH/ART@PLGA/Fe_3_O_4_, PFH/ART@PLGA/Fe_3_O_4_-e and PFH/ART@PLGA/Fe_3_O_4_-eFANPs. From the top to the bottom, the images show DiI-labeled NPs, DAPI-labeled nuclei and the corresponding merged images. **(C)** The phagocytosis of PFH/ART@PLGA/Fe_3_O_4_-eFA and PFH/ART@PLGA/Fe_3_O_4_ NPs by 4T1 cells was observed by flow cytometry. All the error bars represent standard deviation (n = 5).

FA receptors are highly expressed on the surface of tumor cells to meet their needs of rapid proliferation and metabolism (25, 26).Therefore, the modification of NPs with FA to achieve targeted effects is one of the most effective approaches in cancer therapy. Cancer cell and macrophage uptake studies were performed in 4T1 and RAW264.7 cells to evaluate the potential of PFH/ART@PLGA/Fe_3_O_4_-eFA to passively target cancer cells. As shown in **Figure 3A**, the red fluorescence signals of the NPs surrounding the blue fluorescence signals of the 4T1 cell nucleus were weak in the nontargeted group (PFH/ART@PLGA/Fe_3_O_4_-e and PFH/ART@PLGA/Fe_3_O_4_),but the targeted group (PFH/ART@PLGA/Fe_3_O_4_-eFA)showed a strong red fluorescence signal, which indicated that the FA-modified EMs played an important role in the targeting and uptake efficiency of the Nano medicine. As shown in **Figure 3B**, the red fluorescence signals of then on-EM-coated NPs(PFH/ART@PLGA/Fe_3_O_4_)in the intracellular space of RAW264.7 cells were strong, but the EM-coated NPs (PFH/ART@PLGA/Fe_3_O_4_-e and PFH/ART@PLGA/Fe_3_O_4_-eFA) emitted weak red fluorescence signals, indicating that the EM-coated NPs possess the ability to escape endothelial system clearance. To further study the phagocytosis of PFH/ART@PLGA/Fe_3_O_4_ and PFH/ART@PLGA/Fe_3_O_4_-eFA by 4T1 cells, we observed the fluorescence signal intensity by flow cytometry. The results showed that the curve of the PFH/ART@PLGA/Fe_3_O_4_-eFA group shifted significantly to the right compared with that of the PFH/ART@PLGA Fe_3_O_4_ group (**Figure 3C**). PFH/ART@PLGA/Fe_3_O_4_-eFA showed increased uptake by 4T1 cells compared with PFH/ART@PLGA/Fe_3_O_4_ (67.03% vs. 11.41% at 2 h after incubation, 85.25% vs. 29.74% at 4 h after incubation, 92.91% vs. 56.42% at 6 h after incubation). These results suggested that PFH/ART@PLGA/Fe_3_O_4_-eFAcan be efficiently taken up by 4T1 cells. These results make a vital contribution to understand the competitive interaction between FA-modified NPs and non-FA-modified NPs for FA receptors on cancer cells.

### *In vivo* tumor accumulation and biodistribution of PFH/ART@PLGA/Fe_3_O_4_-eFA

**Figure 4:**
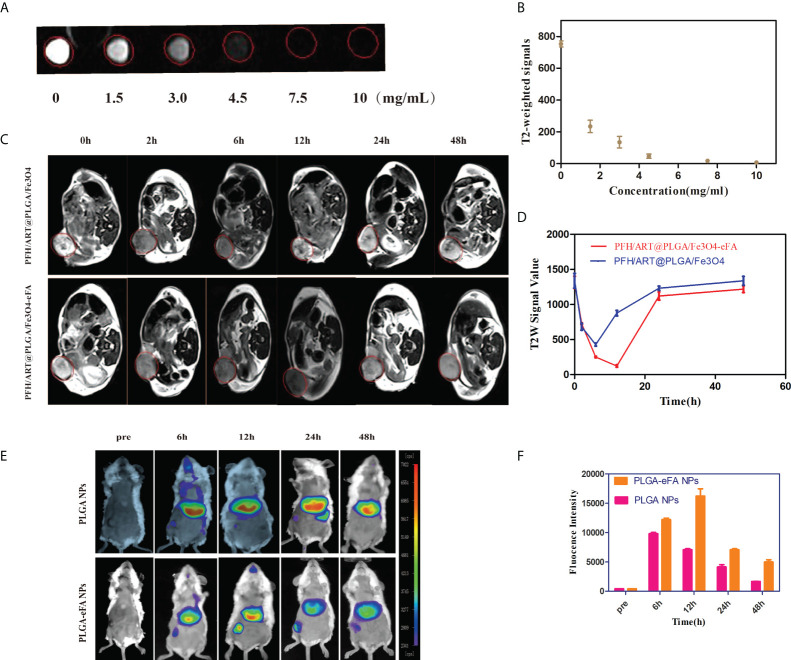
*In vivo* tumor accumulation and biodistribution of NPs. **(A)** *In vitro* MRI signal intensity of PFH/ART@PLGA/Fe_3_O_4_-eFA. *In vitro* MRI images of PFH/ART@PLGA/Fe_3_O_4_-eFA at different concentrations (0, 1.5, 3, 4.5, 7.5, and 10 μg/mL). **(B)** *In vitro* MRI signal intensity curve of different concentrations of PFH/ART@PLGA/Fe_3_O_4_-eFA. **(C)** The MRI T2-weighted imaging signal intensity of 4T1 tumor-bearing mice injected with PFH/ART@PLGA/Fe_3_O_4_ and PFH/ART@PLGA/Fe_3_O_4_-eFA NPs (8mg/kg) captured at different time points after intravenous injection. Red circles highlight the tumor site. **(D)** Quantitative analysis of the MRI T2-weighted imaging signal intensity of breast cancer at different time points after the intravenous injection of PFH/ART@PLGA/Fe_3_O_4_-eFA and PFH/ART@PLGA/Fe_3_O_4_ NPs. **(E)** *In vivo* distribution of fluorescence signals in major organs and tumors at 6 h, 12 h,24 h, and 48 h after intravenous injection of PFH/ART@PLGA/Fe_3_O_4_ and PFH/ART@PLGA/Fe_3_O_4_-eFA. **(F)** Quantitative analysis of the fluorescence of tumors at 6 h, 12 h, 24 h, and 48 h after intravenous injection of PFH/ART@PLGA/Fe_3_O_4_ and PFH/ART@PLGA Fe_3_O_4_-eFA. All the error bars represent standard deviation (n = 5).

We studied the MRI signal intensity of different concentrations of PFH/ART@PLGA/Fe_3_O_4_-eFA *in vitro*. As shown in **Figures 4A, B**, the MRI signal intensity decreased with increasing concentrations of PFH/ART@PLGA/Fe_3_O_4_-eFA *in vitro*. The advantages of FA-modified EMs as carriers to load NPs include the abilities to escape the immune system and target tumors. We further explored the tumor accumulation and biodistribution of the PFH/ART@PLGA/Fe_3_O_4_-eFA. To explore the ability of the PFH/ART@PLGA/Fe_3_O_4_ and PFH/ART@PLGA/Fe_3_O_4_-eFA NPs to be detected by MRI and determine the best time point for the subsequent LIFU irradiation, an *in vivo* T2-weighted MRI study was performed in a mouse model. Nude mice bearing 4T1 xenograft tumors were intravenously injected with PFH/ART@PLGA/Fe_3_O_4_ or PFH/ART@PLGA/Fe_3_O_4_-eFA NPs and imaged at different time points after NPs injection. Significant darkening effects were observed in all the mice after NPs injection, which indicated the ability of the NPs to be imaged. The lowest intensity ratio was observed in the PFH/ART@PLGA/Fe_3_O_4_ group at 6 h after PFH/ART@PLGA/Fe_3_O_4_ NPs injection (**Figure 4C**). In addition, darker tumor areas and longer lasting contrast signals were observed in the tumor regions of mice injected with PFH/ART@PLGA/Fe_3_O_4_-eFA than in the mice injected with PFH/ART@PLGA/Fe_3_O_4_. After the quantitative analysis of all the images, the lowest intensity ratio was observed at 12 h after PFH/ART@PLGA/Fe_3_O_4_-eFA NP injection, which suggests that the highest accumulation of NPs in tumors was achieved at 12 h after PFH/ART@PLGA/Fe_3_O_4_-eFA NPs injection (**Figure 4D**). The *in vivo* MRI data demonstrated that 12 h after PFH/ART@PLGA/Fe_3_O_4_-eFANPs injection could be a good time point for LIFU irradiation. At different time points after NPs injection, the fluorescence intensities of the heart, liver, lungs, spleen, kidneys, and tumors were detected *in vivo* (**Figure 4E**). The fluorescence intensities were enriched in the liver and spleen due to the smaller volume of the tumor than that of the liver and spleen and the low binding efficiency of FA-modified EMs and PFH/ART@PLGA/Fe_3_O_4_.Quantitative analysis (**Figure 4F**) showed that the fluorescence intensities of the tumors in the PFH/ART@PLGA/Fe_3_O_4_-eFA group, which peaked at 12 h, were obviously stronger than those in the PFH/ART@PLGA/Fe_3_O_4_ group. This result suggested that FA-modified EM NPs were “invisible” to the host immune system and tumor NP accumulation was improved, as previously reported (27; 12). Therefore, we determined the best time point for the subsequent LIFU irradiation *in vivo*.

### *In vitro* and *in vivo* PFH phase transition

**Figure 5:**
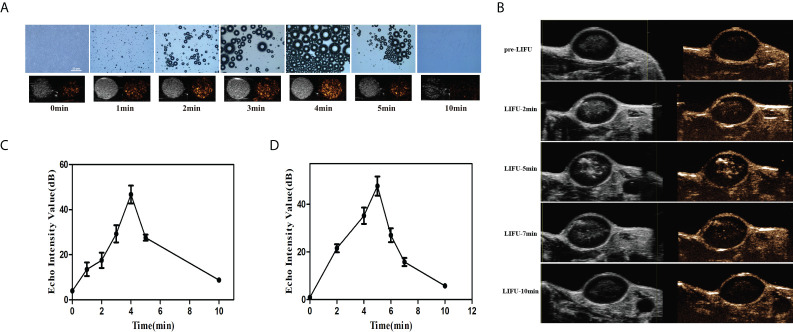
*In vitro* and *in vivo* PFH phase transition. **(A)** *In vitro* optical microscopy images of PFH/ART@PLGA/Fe_3_O_4_-eFA at different time points (pre-irradiation and 1, 2, 3, 4, 5 and 10 min post-LIFU irradiation, from left to right) and ultrasound (US) images captured in contrast-enhanced ultrasound (CEUS) and B-mode (bottom row) of the gas microbubbles generated by the corresponding PFH/ART@PLGA/Fe_3_O_4_-eFA at the same time points. **(B)** *In vivo* echo signal intensity of US images captured in CEUS mode of tumors at different time points (pre-irradiation and 2, 5, 7 and 10 min post irradiation) at 12 h after PFH/ART@PLGA/Fe_3_O_4_-eFA NP injection. **(C)** Quantitative analysis of the echo signal intensity of the US images captured in CEUS mode of NPs *in vitro*. **(D)** Quantitative analysis of the echo signal intensity of US images captured in CEUS mode of tumors *in vivo*. All the error bars represent standard deviation (n = 3).

To confirm that LIFU triggered the phase transformation of the PFH/ART@PLGA/Fe_3_O_4_-eFA NPs and to evaluate the appropriate ultrasound conditions, we exposed PFH/ART@PLGA/Fe_3_O_4_-eFA NPs to LIFU irradiation at 3.0W/m^2^ for 1 min, 2 min, 3 min, 4 min, 5 min and 10 min *in vitro*. The diameter of the NPs observed by optical microscopy gradually increased due to PFH phase transformation after LIFU irradiation (**Figure 5A**). The results showed that the diameter of the NPs peaked at 4 min. The diameter of the NPs decreased rapidly at 5 min and the NPs almost disappeared at 10 min after LIFU irradiation. The echo signal intensity of the US images of NPs captured in CEUS mode gradually increased after LIFU irradiation. As shown in **Figure 5C**, the echo signal intensity peaked at 4 min, decreased rapidly at 5 min and almost disappeared at 10 min, according to quantitative analysis, indicating that the exposure of NPs to LIFU irradiation at 5 min induced NPs collapse due to the liquid–gas phase transition of PFP *in vitro*. Therefore, 3W/m^2^ for 5 min was determined to be suitable conditions for subsequent LIFU irradiation of tumor cells *in vivo*. In this study, the accumulation of NPs at the tumor site peaked at 12 h after PFH/ART@PLGA/Fe_3_O_4_-eFA NPs injection, so we exposed tumors to LIFU irradiation at 3.0 W/m^2^ for 2 min, 5 min, 7 min, and 10 min within the optimal time frame. The results showed that the echo signal intensity of the US images of the tumor captured in CEUS mode gradually increased (**Figure 5B**). As shown in **Figure 5D**, the echo signal intensity peaked at 5 min, decreased rapidly at 7 min and almost disappeared at 10 min, according to quantitative analysis, indicating that exposure of the NPs to LIFU irradiation at 7 min induced NP collapse due to the liquid–gas phase transition of PFH *in vivo*. Therefore, 3W/m^2^ for 7 min was determined to be suitable conditions for subsequent LIFU irradiation of tumors *in vivo*.

### *In vitro* therapy

**Figure 6:**
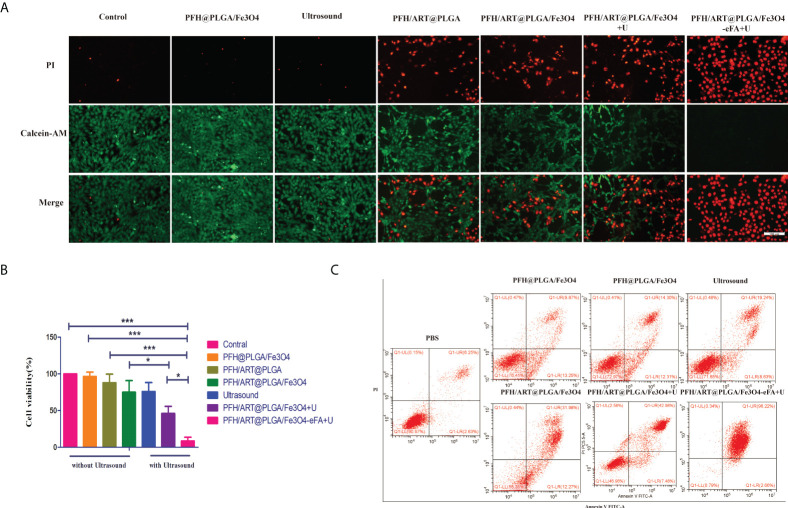
*In vitro* 4T1 cell therapy under different conditions. **(A)** The viability and death of 4T1 cells under different treatment conditions. Laser confocal microscopy images of 4T1 cells stained with calcein-AM/PI.(Green: calcein-AM, representing live cells; red: PI, representing dead cells; the scale bar is 100 nm). **(B)** We investigated the cytotoxicity of the NPs to 4T1 breast cancer cells *in vitro* by using the CCK-8 assay. **(C)** The viability and death of 4T1 cells under different treatment conditions was determined by flow cytometry. Annexin V staining of viable cell membranes and PI staining of dead cell nuclei.High Annexin V and low PI staining indicates early cell death, and high Annexin V and high PI staining indicates late cell death. The levels of significance wereset to values of *p < 0.05, and ***p < 0.001. The data are shown as the mean ± s.d. n = 3.

The viability and death of 4T1 cells under different treatment conditions were studied by CLSM. As shown in **Figure 6A**, there was substantial green fluorescence and almost no red fluorescence in the 4T1 cells in the following groups: the control,LIFU(U) and PFH@PLGA/Fe_3_O_4_ groups, which proved that there was almost no 4T1 cell death. However, the alternate red and green fluorescence signals in the calcein-AM/PI merged images of the 4T1 cells in the PFH/ART@PLGA and PFH/ART@PLGA/Fe_3_O_4_ groups showed a mild cell-killing efficiency. After LIFU irradiation, the red fluorescence signal of the 4T1 cells in the PFH/ART@PLGA/Fe_3_O_4_ group further increased, which proved that triggering drug release by LIFU irradiation could enhance the percentage of 4T1 cell death. The PFH/ART@PLGA/Fe_3_O_4_-eFA+U groups after LIFU irradiation showed stronger red fluorescence signals in the 4T1 cells, indicating that FA-modified EMs improved the efficiency of drug delivery and utilization.Further quantification withthe CCK-8 assay indicated a low cellviability of 5.3% in the PFH/ART@PLGA/Fe_3_O_4_-eFA group after LIFU irradiation compared with other therapeutic groups (**Figure 6B**), suggesting successful *in vitro* LIFU irradiation.Flow cytometry and Annexin V/PI staining were used to further quantify the effect of the different treatments on cell death. As shown in **Figure 6C**, the percentage of cell death was 8.88% in the control group, while in the PFH@PLGA/Fe_3_O_4_ group and PFH/ART@PLGA group, the percentage of cell death was 23.12% and 26.61%, respectively. Compared with PFH/ART@PLGA, PFH/ART@PLGA/Fe_3_O_4_ increased the cell death, and the percentage of cells died was 44.15%.After LIFU irradiation, the proportion of dead cells in the PFH/ART@PLGA/Fe_3_O_4_+U group and PFH/ART@PLGA/Fe_3_O_4_-eFA+U group further increased to 50.46% and 98.88%, respectively, which proved that triggering drug release by LIFU irradiation could enhance the toxic effect of the NPs on 4T1 cells.

**Figure 7:**
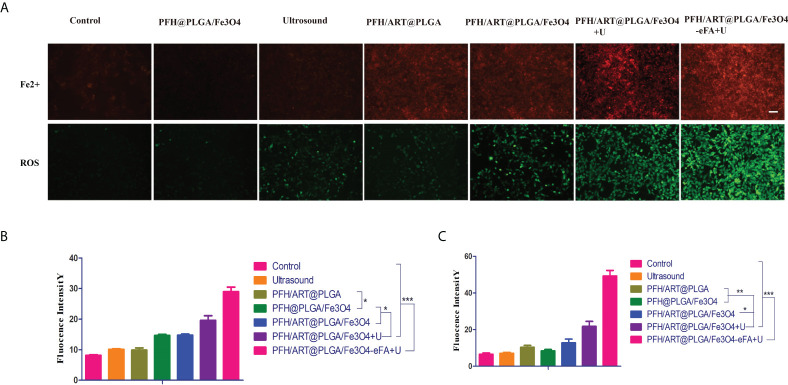
*In vitro* 4T1 cell therapy under different conditions. **(A)** Inverted fluorescence microscopyimages of 4T1 cells were collected to visualize intracellular Fe^2+^ion generation using the Fe^2+^ fluorescent probe ferrorange. Red fluorescence indicates intracellular Fe^2+^ ions in 4T1 cells (upper row). The scale bar is 100 nm. Measurement of intracellular ROS levels with the DCFH-DA probe after different treatments. Green fluorescence indicates ROS levels in 4T1 cells (bottom row).The scale bar is 100 nm. **(B)** Quantitative analysis of the red fluorescence intensity of the intracellular Fe^2+^ ions in 4T1 cells after different treatments. **(C)** Quantitative analysis of the red fluorescence intensity of ROS level in 4T1 cells after different treatments. The levels of significance wereset to values of *p < 0.05, **p < 0.01, and ***p < 0.001. The data are shown as the mean ± s.d. n = 3.

The intracellular Fe^2+^ ion levels were assessed by inverted fluorescence microscopy.As shown in **Figure 7A**, there was almost no red fluorescence inthe 4T1 cells in the control, LIFU, and PFH/ART@PLGA groups, but the red fluorescence in the 4T1 cells in the PFH@PLGA/Fe_3_O_4_ and PFH/ART@PLGA/Fe_3_O_4_ groups, in which the treatments included Fe_3_O_4_, was weak. After LIFU irradiation, the red fluorescence signals in the PFH/ART@PLGA/Fe3O4 group increased from 14.75 to 19.61,which proved that triggering drug release by LIFU irradiation could enhance the level of intracellular Fe^2+^ ions in 4T1 cells. As shown in **Figure 7B**, the red fluorescence signals in the PFH/ART@PLGA/Fe_3_O_4_-rFA+U groups after LIFU irradiation were stronger thanthose inthe PFH/ART@PLGA/Fe_3_O_4_+U groups after LIFU irradiation (28.99 to 19.61), which indicated that FA-modified EMs played an important role inenhancing the levels of intracellular Fe^2+^ ions. It is possible that the low pH value in malignant tumor cells destabilizes the binding of iron to transferrin and allows iron to escape from transferrin Fe^3+^ to Fe^2+^ after more Fe_3_O_4_ enters the 4T1 cells (21). Some studies suggest that increased intracellular levels of Fe^2+^ ions might kill cancer cells through ferroptosis (28). Many studies have reported that ART and its derivatives could lead to cancer cell death through a ferroptosis-dependent pathway (29, 30). Moreover,cancer cell ferroptosisis enhanced by synergistic effects of the cytotoxicity of ART and increased intracellular levels of Fe^2+^ ions (22, 31).Cellular ROS generation was assessed with a ROS-sensitive fluorescent probe (DCFH-DA). The green fluorescence emitted by DCFH-DA in the 4T1 cells in different treatment groups appeared after incubation with the treatments for 2 h. As shown in **Figure 7A** (bottom row), there was almost no green fluorescence signal emitted by DCFH-DA in the 4T1 cells in the control, LIFU, PFH/ART@PLGA and PFH@PLGA/Fe_3_O_4_ groups, but the green fluorescence signal emitted by DCFH-DA in the 4T1 cells in the PFH/ART@PLGA/Fe_3_O_4_ group was weak. After LIFU irradiation, the green fluorescence signal in the PFH/ART@PLGA/Fe_3_O_4_ group increased from 12.97 to 21.75 (**Figure 7C**),which proved that triggering drug release by LIFU irradiation could enhance the level of ROS in 4T1 cells. As shown in **Figure 7A, B**, the red fluorescence signals in the PFH/ART@PLGA/Fe_3_O_4_-rFA+U groups after LIFU irradiation were stronger than those in the PFH/ART@PLGA/Fe_3_O_4_+U groups after LIFU irradiation (21.75to 49.27), which indicated that FA-modified EMs played an important role in enhancing the levels of ROS in 4T1 cells.After LIFU irradiation, the proportion of dead 4T1 cells,the intracellular concentration of Fe^2+^ ions, and the level of ROS in the PFH/ART@PLGA/Fe_3_O_4_-eFA+U group were highest of those in the all the groups (**Figures 7A–C**), indicating that intracellular Fe^2+^ions in the cancer cells played a critical role in tumor cell ferroptosis by enhancing the generation of ROS.

### *In vivo* antitumor efficacy

**Figure 8:**
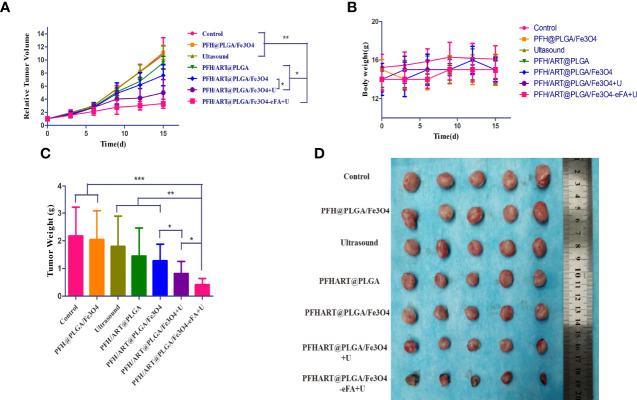
*In vivo* treatment. **(A)** The growth curve of murine *in situ* breast cancer tumors under different treatment conditions. **(B)** The body weight curves of the mice under different treatment conditions. **(C)** Quantitative analysis of the weights of the tumorsin mice under different treatment conditions. **(D)** Tumors harvested from each group at 15 d post treatment. The levels of significance wereset to values of *p < 0.05, **p < 0.01, and ***p < 0.001. The data are shown as the mean ± s.d. n = 5.

The *in vivo* antitumor efficacy was then evaluated using 4T1 breast tumor-bearing nude mice. The tumor-bearing mice were randomly divided into seven groups with six mice in each group: the control group, PFH@PLGA/Fe_3_O_4_ group, ultrasound group, PFH/ART@PLGA group, PFH/ART@PLGA/Fe_3_O_4_ group,PFH/ART@PLGA/Fe_3_O_4_+U group, and PFH/ART@PLGA/Fe_3_O_4_-eFA+U group. **Figure 8A** shows the tumor sizes as a function of time. The tumor growth volume curves show that the tumors in the control group, PFH@PLGA/Fe_3_O_4_ group and ultrasound group grew rapidly, indicating that Fe_3_O_4_ or ultrasound alone could not inhibit tumor growth. The slight tumor inhibition in the PFH/ART@PLGA group and PFH/ART@PLGA/Fe_3_O_4_ group indicated that ART and the combination of ART and Fe_3_O_4_ could not effectively suppress tumor growth.The tumors in the mice treated with PFH/ART@PLGA/Fe_3_O_4_+U showed markedly smaller volumes and weights than those in the mice treated with PFH/ART@PLGA/Fe_3_O_4_ due to the increased release of ART and Fe_3_O_4_ after LIFU irradiation (**Figure 8A, C**).The tumors in the mice treated with PFH/ART@PLGA/Fe_3_O_4_-eFA+U had markedly smaller volumes and weights than those in the mice treated with PFH/ART@PLGA/Fe_3_O_4_+U due to the preferential accumulation of FA-modified EMs at the tumor sites.Among all the groups, the PFH/ART@PLGA/Fe_3_O_4_-eFA+U group exhibited the greatest tumor regression over time due to the synergetic antitumor effects exerted by triggering Fe_3_O_4_ and ART release with LIFU irradiation after the abundant accumulation of NPs in the tumor.The volumes and weights of the excised tumors were very consistent with those measured in living mice (**Figure 8A, C**). Here, the body weight of the tumor-bearing mice showed no obvious decrease, indicating minimal biotoxicity and no drug leakage during NP delivery and LIFU irradiation (**Figure 8B**).

**Figure 9:**
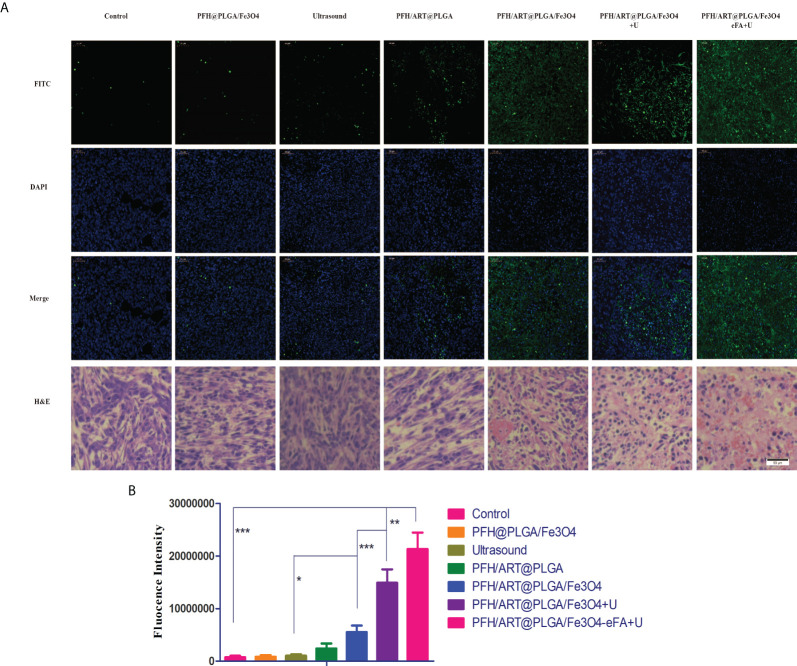
*In vivo* treatment. **(A)** H&E and TUNEL staining of the tumors15d after the different treatments.Nuclei and apoptotic cells were stained blue and green, respectively. **(B)** Quantitative analysis of the fluorescence intensity of TUNEL staining. Scale bars indicate 50 µm. The levels of significance wereset to values of *p < 0.05, **p < 0.01, and ***p < 0.001. The data are shown as the mean ± s.d. n = 5.

*In situ* terminal-deoxynucleotidyltransferase-mediated nick end labeling (TUNEL) analysis and H&E staining demonstrated that of all the treatment groups,the highest degree ofcell death was observed in the PFH/ART@PLGA/Fe_3_O_4_-eFA+U group, which was also consistent with the highest antitumor efficacy of the PFH/ART@PLGA/Fe_3_O_4_-eFA+U treatment (**Figure 9A**). After LIFU irradiation, the quantitative analysis showed that the fluorescence intensity at the tumor site in the PFH/ART@PLGA/Fe_3_O_4_+U group was greatly enhanced (**Figure 9B**),which proved that triggering drug release by LIFU irradiation could increase tumor cell death.The quantitative analysis showed that the fluorescence intensity at the tumor site in the PFH/ART@PLGA/Fe_3_O_4_-eFA+U groups after LIFU irradiation was stronger than that in the PFH/ART@PLGA/Fe_3_O_4_+U groups after LIFU irradiation (**Figure 9B**), which indicated that FA-modified EMs could increase tumor cell death *via* the accumulation of NPs at the tumor site.

## Conclusions

In this study, we constructed FA-modified EMs into vesicles and encapsulated them onto PLGA loaded with phase-changeable PFH, magnetic Fe_3_O_4_ and ART. PFH/ART@PLGA/Fe_3_O_4_-eFA was exposed to LIFU irradiation to induce PFH phase transition and NPs collapse,which promoted the release of ART and Fe_3_O_4_ *in vitro*. The high efficacy of this approach is ascribed to the synergistic effect of intracellular Fe^2+^ ions and ART, which played critical roles in inducing tumor cell ferroptosis by enhancing ROS generation *in vitro*. We demonstrated that FA-modified EM NPs could enhance the targeting and accumulation of the PFH/ART@PLGA/Fe_3_O_4_-eFA NPs at the tumor site *in vivo*.After PFH/ART@PLGA/Fe_3_O_4_-eFA injection and LIFU irradiation at 3 W/m^2^ for 7 min, tumor growth was completely suppressed *in vivo*. Therefore, combining PFH/ART@PLGA/Fe_3_O_4_-eFA and LIFU irradiation enhanced the therapeutic efficacy of drugs with poorwater solubility and provided a promising approach for their delivery for cancer treatment in clinic.

## Data availability statement

The original contributions presented in the study are included in the article/supplementary files. Further inquiries can be directed to the corresponding authors.

## Ethics statement

The animal study was reviewed and approved by the Animal Ethics Committee of Chongqing Medical University.

## Author contributions

HR and HT contributed to conceptualization and data curation. XW and PL contributed to construct EM-Coated PLGA-NPs and cells experiments. YZ, MZ and JW contributed to animals experiments. XJ contributed to analysis and investigation. HT contributed to write–original draft preparation and write–review and editing. YS contributed to supervision. All authors discussed the results and commented on the manuscript. All authors contributed to the article and approved the submitted version.

## Funding

This work was supported by Basic Public Welfare Research Program Project of Zhejiang Province (grant no. LSY19H180007), the Health Department of Zhejiang Province (grant no. 2020KY090), the National Natural Science Foundation of China (grant no.31901045, 21877083, 81871365, 81760317).

## Conflict of interest

The authors declare that the research was conducted in the absence of any commercial or financial relationships that could be construed as a potential conflict of interest.

## Publisher’s note

All claims expressed in this article are solely those of the authors and do not necessarily represent those of their affiliated organizations, or those of the publisher, the editors and the reviewers. Any product that may be evaluated in this article, or claim that may be made by its manufacturer, is not guaranteed or endorsed by the publisher.
